# Investigating Apple Rubbery Wood Virus 2: HTS-Based Detection in Hungary and Involvement of sRNA-Based Regulation Processes During Its Infection

**DOI:** 10.3390/v17101394

**Published:** 2025-10-20

**Authors:** Almash Jahan, Éva Várallyay

**Affiliations:** Genomics Research Group, Department of Plant Pathology, Institute of Plant Protection, Hungarian University of Agriculture and Life Sciences, Szent-Györgyi Albert Street 4, 2100 Gödöllő, Hungary; almashjahan010@gmail.com

**Keywords:** apple, rubbery wood, ARWV2, pear, miRNA pattern, lignin synthesis, phenylpropanoid pathway

## Abstract

Pomme fruits are propagated vegetatively, thereby facilitating frequent viral transmission. The causative agent of apple rubbery wood disease, apple rubbery wood virus 2 (ARWV2), can infect apple and pear. The branches of ARWV2-infected, symptomatic trees are flexible due to the decreased lignification of the xylem. In this research, we reanalysed our small RNA (sRNA) HTS datasets to survey the presence of ARWV2 in Hungary. Validation of HTS using RT-PCR revealed infection in several cultivars. The following RT-PCR-based survey revealed the infection of 17 trees, including not only apple, but also pears, one quince, and a rootstock, without showing any rubbery wood symptoms. Analysis of the sRNA datasets allowed us to profile the sRNA pattern of ARWV2-infected and non-infected trees, and characterise the differential expression pattern of vsiRNAs, sRNAs, and miRNAs targeting the lignin biosynthetic pathway. The results confirmed that the gene-expression changes in the genes that regulate lignification cannot be directly correlated with the presence of the virus, which can explain its frequent latent presence. The variable titre and sequence of the virus, and mixed-infection status of the trees, make its reliable diagnostics challenging, which could be achieved as a result of further research.

## 1. Introduction

Pome fruits are an important source of fibres and vitamins, grown and consumed all over the world. Apples (*Malus* spp.) and pears (*Pyrus* spp.) are the most widely and commercially cultivated species of the *Malus* genus throughout temperate regions of the World. Their production reached 95.8 and 26.3 million metric tons in 2022 (https://worldpopulationreview.com, accessed on 1 August 2025,), while quince (*Cydonia* spp.) is produced in a much lower quantity (0.68 million metric tons in 2023). Apple and pear production is adversely impacted by several viral diseases that can lead to reduced yield, poor fruit quality, and shortened tree lifespan, while quince seems to be more resistant to viral infection. Viruses with the biggest impact on apple and pear are apple chlorotic leaf spot virus (ACLSV), apple mosaic virus (ApMV), apple stem grooving virus (ASGV), and apple stem pitting virus (ASPV). Infection with these viruses often remains latent but may cause symptoms such as chlorosis, leaf distortion, graft incompatibility, and diminished tree vigour. Besides these viruses, whose presence is under regulation in the propagating material, apple can be infected with recently described viruses: citrus concave gum-associated virus (CCGaV) [1], apple luteovirus 1 (ALV-1) [2], and viruses causing the disease of the wood of the apple tree.

Apple rubbery wood disease (ARWD) was first observed on the Lord Lambourne cultivar in 1935 in England [3], showing flexible limbs and atrophy of the vascular tissue. Infected trees exhibit flattened shoots and limbs, particularly on 2- to 3-year-old growth. As branches age, flattening becomes more pronounced, leading to deep furrows. The fruit yield of the diseased tree can drop to 30% in apple and 50% in pear [4]. In 2018, the viral agents of the ARWD, two RNA viruses: apple rubbery wood virus 1 and 2 (ARWV1 and 2), were identified and characterised [5], and ARWV2 (Rubodvirus prosserense) presence has been proven to be associated with ARWD [6,7]. Since then, ARWV2 has been found in pear, indicating a broader host range [6,8].

In the past, symptoms of ARWD have been found very frequently, but as a result of reliable certification programmes, the disease has been successfully eliminated from commercial rootstocks. The virus reemerged, or has started to be detected, when HTS was introduced to use for viromes studies. From that time, it has been described worldwide, but as a latent virus, whose concentration in the trees fluctuates and whose RT-PCR detection results in uncertainties several times.

ARWV2 has been reported in various parts of the world, including the UK, Germany and USA, where it was originally detected, and has been found in China [9], Brazil [10], South Africa [11], Italy [12], Turkey [13], and, more recently, in the Czech Republic [14] and Iran [15]. Other than the virus identification, in the absence of directed studies, there is very limited knowledge about how the environment affects the viral symptoms. Additionally, the investigated ARWV2-infected trees are usually coinfected with other widely distributed latent viruses; it is very difficult to connect the observed symptoms directly to ARWV2 presence.

ARWV2 is a tripartite, negative-sense single-stranded RNA virus belonging to the *Rubodvirus* genus of the *Phenuiviridae* family. Complementary RNA (cRNA) of each genomic RNA: RNA1 (large; L), RNA2 (medium; M), and RNA3 (small; S) has a single open reading frame (ORF), encoding an RNA-dependent RNA polymerase (RdRp), a movement protein (MP), and a coat protein (CP), respectively. Some ARWV2 isolates possess two separate M and S segments, named Ma, Mb, Sa, and Sb. Sequences of the Ma and Mb segments share approximately 66%, while the Sa and Sb segments share 56% nucleotide identity [6]

In apple trees suffering from ARWD, the lignin content of the xylem is altered, and this is why the trees exhibit the typical symptom of the disease: rubbery wood [16]. Gene expression changes in the infected trees were characterised, revealing altered expression of the key enzymes on the phenylpropanoid pathway. Although none of the virus-specific sRNAs targeted any of the genes of this pathway, an increased number of the vsiRNAs targeting the phenylalanine ammonia lyase (PAL) genes had been identified, and their inhibiting effect by an unknown mechanism has been suggested [16].

Lignin is a phenolic polymer whose presence in the woody secondary cell wall makes the carbon feedstock, deposited in the plant biomass, difficult to achieve. To replace the cost-intensive chemical treatment before cellulose hydrolysis, the lignin content of plants has been genetically engineered [17,18]. Analysing the chemical composition of the ARWV2-infected young grafts showed the same type of decrease in the cell wall phenolic compounds and increased the digestibility of the fibres [16].

In our previous study, investigating the viromes of apple trees using HTS, we found the widespread presence of ALV-1 and CCGaV in Hungary and the Czech Republic [19]. Revisiting these published datasets using updated virus lists as a reference, the presence of ARWV1 and 2 [14] in the Czech Republic and ARWV2 in Hungary [20] has been revealed.

In this study, we present a detailed description of the ARWV2 diagnostics using sRNA HTS, and the detailed results of a small-scale RT-PCR survey of apple, pear, quince, and rootstocks growing in Hungary. To gain further insight into the molecular mechanism of this virus-caused disease, we explored changes in the sRNA pattern of the ARWV2-infected apple host, aiming to map the possible role of sRNA-based regulation response mechanisms in the development of ARWD.

## 2. Materials and Methods

### 2.1. Origin of the Samples, RNA Extraction, and sRNA HTS

Asymptomatic leaf samples originating from 114 trees: 101 apples (*Malus domestica*), 9 pears (*Pyrus communis*), 1 quince (*Cydonia oblonga*), and 4 rootstocks, were collected from production orchards and germplasm collections, from different geographical locations in Hungary between 2015 and 2021 (Table A1, Figure S1 and Table S1).

Leaves were sampled from four different branches per tree and used for RNA extraction using an optimised protocol [21], based on the CTAB method of Gambino [22]. The quality and quantity of the RNAs were tested by agarose gel electrophoresis and 260/280 photometry on a NanoDrop machine (Thermo Fisher Scientific, Waltham, MA, USA). For sRNA high-throughput sequencing (sRNA HTS), RNA pools representing either individual trees or a mixture of ten trees (in the case of the 16_apple) were prepared by combining equal amounts of total RNA, as detailed previously [19] (Table A1). sRNA libraries were constructed from purified sRNAs using the TruSeq sRNA Library Preparation Kit (Illumina, San Diego, CA, USA), following an in-house modified protocol [21]. In total, 22 sRNA libraries were prepared and sequenced using a single-index setup on a HiScanSQ platform by UD-Genomed (Debrecen, Hungary), employing 50 bp single-end sequencing. The corresponding FASTQ files have been deposited in the NCBI GEO database under accession numbers GSE205183 and GSE306190.

### 2.2. Bioinformatic Analysis of sRNA Reads for Virus Diagnostics

Bioinformatics analysis was performed using CLC Genomics Workbench version 20.0.4 (Qiagen, Hilden, Germany) [23] (Table S2). Following trimming and quality control, the reads were directly mapped to the reference genome of ARWV2 (NC_055533-537) and were counted with and without redundancy (using the map to the reference command, allowing 1 mismatch). The number of normalised reads (read/1 million reads—RPM) was calculated as the ratio of mapped redundant reads to the number (in millions) of total sequenced reads (Table S3). Based on the optimised bioinformatic analysis, where we used sRNA HTS for viromes determination of woody plants [21], we set three criteria as the limit of infection: (i) the presence of one virus-derived contig, (ii) the presence of higher than 200 RPM, and (iii) larger than 60% coverage of the viral genome. The size distribution of the ARWV2-derived reads was generated from the QC report of CLC.

### 2.3. Validation of the Presence of ARWV2 (cDNA and RT-PCR)

RNA extracts of the trees (sRNA HTS tested or tested as a survey) were reverse transcribed using the RevertAid First Strand cDNA Synthesis Kit (Thermo Fisher Scientific, Waltham, MA, USA) using random primers according to the manufacturer’s instructions. The quality of the cDNA was tested by amplifying a part of the *Malus domestica* actin gene using Phire DNA Polymerase (Thermo Fisher Scientific). RT-PCR reactions amplifying viral sequences were performed using the Q5 High-Fidelity DNA Polymerase (New England Biolabs, Hitchin, UK). Primers for the actin test and ARWV2 detection are listed in Table S4a,b. PCR products intended for Sanger sequencing were cut from the agarose gel and purified using a GeneJET Gel Extraction Kit (Thermo Fisher Scientific). The products were Sanger-sequenced (Eurofins BIOMI KFT., Godollo, Hungary) after cloning into the CloneJET vector (Thermo Fisher Scientific). Sequences of the clones were deposited in NCBI GenBank: PX052001-PX052011 (Table S5).

### 2.4. Phylogenetic Analysis of the ARWV2 Strains

The phylogeny of the ARWV2 partial sequences (segment L, segment M, and segment S) was analysed using Geneious Prime^®^ 2022.1.1 (Biomatters, Auckland, New Zealand). The alignment was performed using the in-built MUSCLE algorithm, while the phylogenetic tree was built based on maximum likelihood phylogenies, using the Tamura-Nei genetic distance model, with 1000 bootstrap values. ARWV2 sequences used in the phylogenetic analyses are listed in the Table S6. The pairwise identity matrix of their nucleic and amino acid sequences, exported from the multiple alignment prepared as described above, is detailed in Table S7.

### 2.5. Bioinformatic Analysis of sRNA Reads for sRNA Profiling

For the detailed sRNA analysis, we analysed four ARWV2-infected and three non-infected sRNA libraries. Differentially expressed miRNAs were analysed by the CLC, including the principal component analysis (PCA), and miRNAs with the highest change were collected (Table S8). After mapping the sRNA reads to ARWV2, both the mapped and unmapped reads were mapped to the mRNAs of the phenylpropanoid genes of the lignin pathway, which were shown to be altered during ARWV2 infection [16]. The sRNAs targeting these genes were extracted and counted, and their patterns in the infected and non-infected apple libraries were statistically compared (Table S9).

### 2.6. RT-qPCR for Expression Analysis of PAL

The differential expression of the apple phenylalanine ammonium-lyase (PAL 1C) was quantified using RT-qPCR. Before cDNA synthesis, total RNA originating from the single trees was treated with DNase I to remove any genomic DNA using the Turbo DNase-Free Kit (Invitrogen by Thermo Fisher Scientific). The DNase-treated RNA was then reverse-transcribed into cDNA using Applied Biosystems™ High-Capacity RNA-to-cDNA™, cDNA Reverse Transcription PCR Kits, including RNase Inhibitor. Specific primers for RT-qPCR were designed by using Primer Blast (website https://www.ncbi.nlm.nih.gov/tools/primer-blast/, accessed on 1 August 2025) software using the apple genome as a reference. RT-qPCR reactions were carried out using the EvaGreen Master Mix (Applied Biosystems, Waltham, MA, USA) on Roche Lightcycler ^®^ 96. See Table S3 for the details of the primers.

Apple actin and ubiquitin genes were used as internal reference genes to normalise gene expression levels. Each qPCR reaction was performed in three technical replicates. Relative expression levels were calculated using the ΔΔ*C*_t_ method of the Roche Lightcycler instrument software. PAL 1C expression was normalised against both reference genes to ensure accurate and stable quantification. Statistical analysis was performed via one-way ANOVA. Post hoc analysis was performed using Tukey’s test and marked using a compact letter display (CLD).

## 3. Results

### 3.1. ARWV2 Is Present in the Apple Trees Growing in Hungary

To investigate the possibility of ARWV1 and ARWV2 infection in apple trees grown at various locations in Hungary (Figure S1), we reanalysed the sRNA datasets prepared during our previous study (Table A1 and Table S2). While we have not found any hints (mapped contigs or sRNA reads) for the presence of ARWV1, we found a mark of infection with ARWV2 in several libraries (Table S2) [20]. In the investigated dataset, we found only one ARWV2-derived contig, present in one of the libraries (7_Freedom), originating from the Sa-segment, whose coverage by ARWV2-derived sRNAs was 92% [20]. According to the limits we set, presence of ARWV2 was detected in five out of 22 libraries: 2_SZH, 7_Freedom, 12_RosmertaSz, 16_apple, and 18_AP2. Beside these hits coverage of the ARWV2 genome was higher than 60% in several other libraries, and lowering the threshold limit of the virus infection to 50% coverage of at least L and one M or S segments, showed ARWV2 infection in additional 10 libraries: 1_Zsz, 3_SS, 4_idared, 8_RosmertaI, 11_CordeliaI, 13_HesztiaSz, 14_ArtemiszSz, 15_CordeliaSz, 18_ReglindisAP2, and 22_E3 (Table S2).

Size distribution of the sequenced virus-mapped reads could reflect their viral origin, so this feature was also investigated (Figure S2). We found that the ARWV2-derived sRNAs were 24 nt in most cases, except in the 7_Freedom library, where most of the ARWV2-derived sRNAs were 21–22 nt long (Figure S2). High abundance of 22 nt long ARWV2-derived sRNA could also be detected in 18_ReglindisAP2.

**Figure 1:**
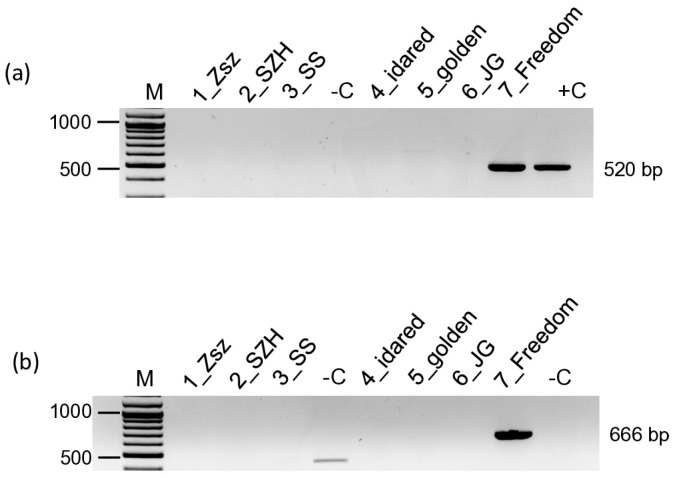
RT-PCR-based test to detect the ARWV2. (**a**) L and (**b**) Ma segments in the samples, which were used for sRNA HTS-based virus diagnostics. M is the 100 bp+ GeneRuler of Thermo Scientific. +C is the positive, while –C is the negative control.

Adjusting thresholds alone does not tell anything about the real infection, so we carried out an RT-PCR-based survey to detect L, Ma, and Sa segments in the same RNA extracts that were used for the sRNA library preparation. For the amplification, we considered using primers from the original study of Rott and colleagues [6]. Before the test, we compared sequences of these primers to the consensus sequence prepared from the sRNA reads. The ARWV2 reads covered the part of the genome where the primers were designed only in the case of the 7_Freedom library, where the primers for the L segment amplification seemed ok, but showed several mismatches in the case of the primers for the Ma segment amplification. For this role, we designed a new primer (Table S3). In these tests, we could amplify L and Ma segments only in the 7_Freedom sample (Figure 1) [20].

These amplified parts were cloned and sequenced (GenBank accession numbers: PX052010-11), and their phylogeny was analysed (Table S5). Coverage of the L segments in the other libraries was usually higher than 40%, and the RPM of the L segment-derived sRNA reads varied from 35 to 128 (lowest in 21_ReglindisE2 and the highest in 16_apple). There were only three libraries, where these limits have not been reached—17_ReglindisAP1, 19_RemoAP3, and 20_ReglindisE1 (10.5, 1.4, and 3.5 RPM)—marking these libraries free from the presence of ARWV2 L segment. RPM of the Ma or Mb-derived sRNAs was even lower (below 10 in the three, above-mentioned, “ARWV2-free” libraries) and was the highest in 7_Freedom (188), where we were able to detect this segment.

**Figure 2:**
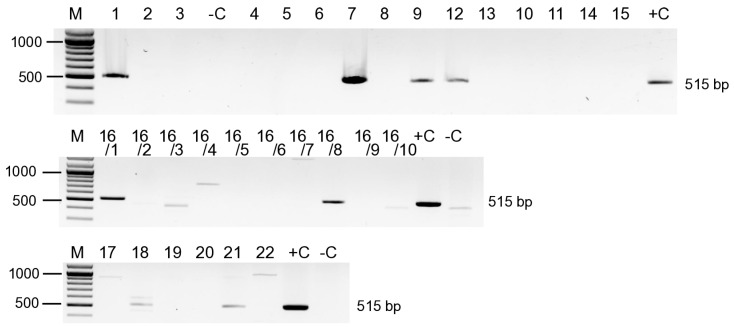
RT-PCR-based test to detect the ARWV2 Sa segment in the samples, which were used for sRNA HTS-based virus diagnostics. Numbers of the samples refer to the sRNA library numbers. (1_Zsz, 2_SZH, 3_SS, 4_idared, 5_golden, 6_JG, 7_Freedom, 8_RosmertaI, 9_HesztiaI, 10_ArtemiszI, 11_CordeliaI, 12_RosmertaSz, 13_HesztiaSz, 14_ArtemiszSz, 15_CordeliaSz, 16_apple, 7_ReglindisAP1, 18_ReglindisAP2, 19_RemoAP3, 20_ReglindisE1, and 21_RemoE2, 22_RemoE3). Individuals in the 16_apple, when RNA of 10 individuals were used for sRNA library preparation in combination, were tested individually (16/1 Ozark gold, 16/2 Jonagold, 16/3 Florina, 16/4 Jim Brian, 16/5 Jeasymac, 16/6 Jonica, 16/7 Red Elstar, 16/8 Regal prince, 16/9 Redwinter, 16/10 Top spur). M is the 100 bp+ GeneRuler of Thermo Scientific. +C is the positive, while –C is the negative control.

CP of ARWV2 is encoded on the S segment (Sa, or Sb), so we expected that the titre of this segment in the “ARWV2 positive” libraries could reach the level of the RT-PCR detection limit in more samples, even if the number of the S segment-derived sRNAs was low (RPM varied between 7 and 316). Amplification of the Sa segment by RT-PCR, using primers designed by considering the sequences of the sRNA reads, validated the presence of the virus in seven libraries: 1_ZSz, 7_Freedom, 9_HesztiaI, 12_RosmertaSz, 16_apple (two individuals), 18_ReglindisAP2, and 21_RemoE2 (Figure 2).

### 3.2. ARWV2 Infection Has Been Found in Pears and Quince

**Figure 3:**
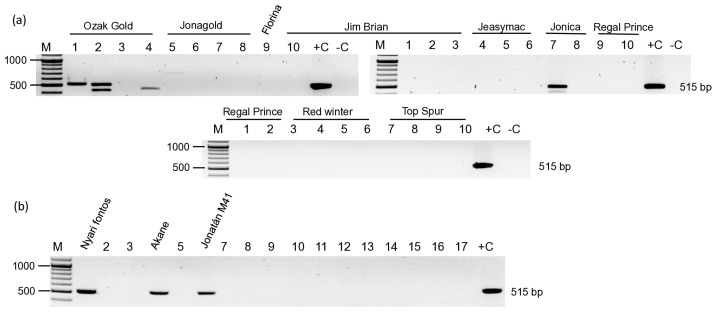
RT-PCR-based test to detect the ARWV2 Sa segment in the samples collected in 2019 at Erd in the (**a**) Old, certified stock collection, and (**b**) Old cultivar collection. M is the 100 bp+ GeneRuler of Thermo Scientific. +C is the positive, while –C is the negative control. Cultivar names of the positive samples are indicated above the photo. The negative cultivars on panel B are 2/Téli arany parmen, 3/Fertődi téli, 5/Starking Nm. 251, 7/Jonagold (12/5Ny), 8/Mutsu, 9/Idared, 10/Gloster, 11/Freedom, 12/Elstar, 13/Florina, 14/Charden, 15/Jerseymac, 16/Red Elstar, and 17/Regal Prince.

**Figure 4:**
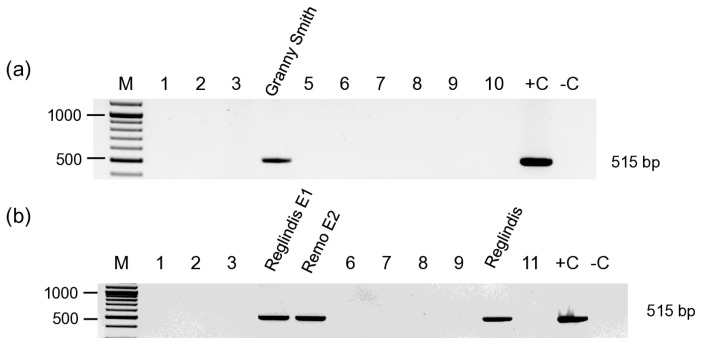
RT-PCR-based test to detect the ARWV2 Sa segment in the samples collected in (**a**) in the new apple cultivar collection in 2020 at Erd, (**b**) in a production garden in 2020 at Olcsvaapáti. M is the 100 bp+ GeneRuler of Thermo Scientific. +C is the positive, while –C is the negative control. Cultivar names of the positive samples are indicated above the photo. The negative cultivars on panel A are 1/Idared, 2/Jonathan M.41, 3/Golden Reiders, 5/Braeburn, 6/Mutsu, 7/Regal Prince, 8/Florina, 9/Red Elstar, 10/Tenroy. The negative cultivars on panel B are 1/Reglindis AP1, 2/Reglindis AP2, 3/Remo AP3, 6/Remo E3, 7/Rebella, 8/Remo, 9/Relinda, and 11/Renora.

As we have found ARWV2 infection in several apple trees, we tested the presence of this virus in the samples, which were collected from pome fruits between 2019 and 2021, to test for the presence of regulated viruses in an old cultivar stock collection, a new germplasm collection at Erd, and in a production orchard in Olcsvaapáti. Based on our previous experience, for the RT-PCR-based survey, we used the diagnostic primers designed to detect the Sa segment. Testing 83 trees, including 8 pears, 4 rootstocks, and 1 quince, we found the presence of ARWV2 in 17 cases (Table S4 and Figure 3 and Figure 4) [20]. In the old collection, two Ozak Gold and one individual of Jonica, Nyári fontos, Akane, and Jonatán M41 were found to be infected (Figure 3).

Some of these trees were tested in 2017, and their RNA was included in the 16_apple library. The first Ozak Gold tree was tested ARWV2 positive both times, while the Jonica was negative in 2017, and the Regal Prince was positive in 2017 and found to be negative in 2019. In the upcoming years, in 2020 and 2021, we tested new stock collections at Erd, when, in addition to apple, pear, quince, and rootstock samples were also collected (Table S4). In this survey, five apple trees: Granny Smith, two Reglindis, Remo and Golden Reinders, one quince (Bereczki cultivar), four pears: Pachams Triumph, Tongre, Vilmos, and Piros Vilmos, and one Ba-29 rootstock were found to be ARWV2 infected (Figure 4) [20].

In the production garden at Olcsvaapáti, some trees tested in this survey in 2020 were the same that were sampled three years before. In 2017, Reglindis AP2 and Remo E3, while in 2020, Reglindis E1 and Remo E2 were tested ARWV2 positive by RT-PCR.

### 3.3. Phylogenetic Analyses of the ARWV2 Atrains of Hungarian Origin

**Figure 5:**
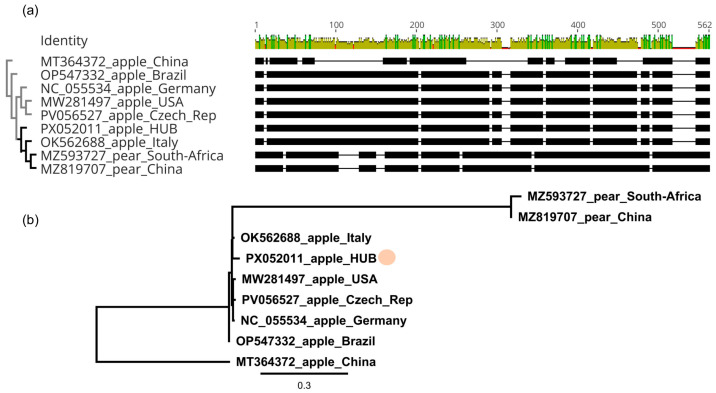
Sequence comparison of the ARWV2 partial L segment. (**a**) multiple alignment, (**b**) phylogenetic analysis of the sequence of the Hungarian variants and the variants present in the NCBI GenBank (see Table S6 for their origin and details). For the multiple alignment MUSCLE algorithm, for the phylogenetic analysis, the neighbour joining method with the Tamura–Nei model, with 1000 bootstrap, was used. The sequence of ARWV2 variants sequenced in China (MT364372) was used as an outgroup. The dot marks the HU variant.

During our surveys, we have found ARWV2 infection in several trees and were able to amplify the L, Ma, and Sa segments from the Freedom cultivar, and the Sa segment from 23 of them. These amplified genome segments were cloned and sequenced (GenBank accession numbers: PX052001-PX052011) and were used for comparison and phylogenetic analysis. The partial sequence of the RdRp encoding L segment was 97.4% identical to the reference genome. Multiple sequence alignment and phylogenetic analysis of the same part of the ARWV2 variants available at the GenBank (Table S6) showed that the sequences from the pear (originating from China and from South Africa) clustered separately, because of the presence of characteristic indels (Figure 5).

The closest neighbour of the Hungarian ARWV2 variant was the variant originating from Italy (OK562688), sharing 97.4% identity (Table S7A).

**Figure 6:**
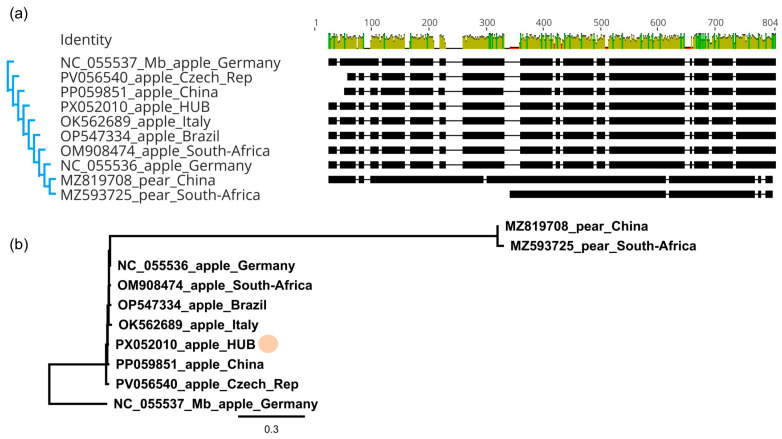
Sequence comparison of the ARWV2 partial Ma segment. (**a**) multiple alignment, (**b**) phylogenetic analysis of the sequence of the Hungarian variants and the variants present in the NCBI GenBank (see Table S6 for their origin and details). For the multiple alignment MUSCLE algorithm, for the phylogenetic analysis, the neighbour joining method with the Tamura–Nei model, with 1000 bootstrap, as a tool in Geneious Prime, was used. The reference sequence of the ARWV2 Mb (NC_055537) was used as an outgroup. The dot marks the HU variant.

The sequence of the M segment of the Hungarian variant, encoding the MP, showed the closest similarity (99,2%) to the variant sequenced in an apple in China (PP059851). Similarly to the L segment, sequences of the Ma segment in the investigated range contained indels, which were characteristic of the host species (Figure 6a) and ARWV2 variants sequenced from pear clustered separately (Figure 6b).

Out of the 23 sequenced partial Sa segments, 9 were different (Table S5). The most abundant HUB strain was present in 11 of the trees, in different cultivars of apple and pear, and it was found at all of the sampled geographical locations. The second abundant strain, HUH, was found in quince, pear, and a rootstock only at Erd, while HUC was found in two different apple cultivars, growing at different locations. In addition to apple, HUB and HUI have also been found in pear. The other variants were found only once. However, we sequenced nine variants; they were more than 98% identical. The amino acid sequence of the partial CP encoded by the HUA, HUB, HUG, and HUI was identical and shared higher than 98% identity with the CP encoded by the other variants and the reference strain. For the phylogenetic analyses, we used ARWV2 sequences in the NCBI GenBank representing variants sequenced from different species at different parts of the world (Table S6).

**Figure 7:**
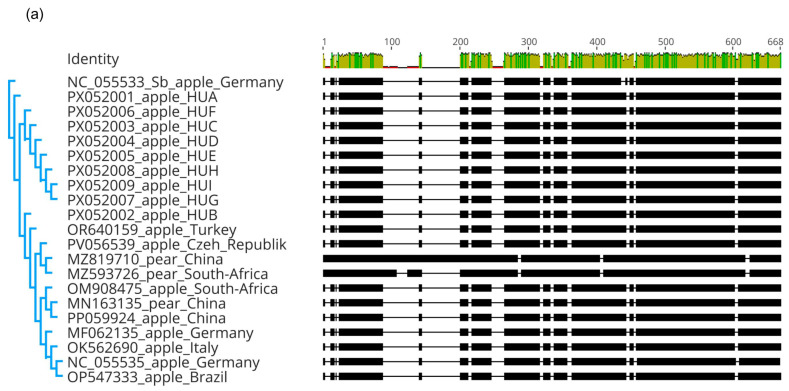

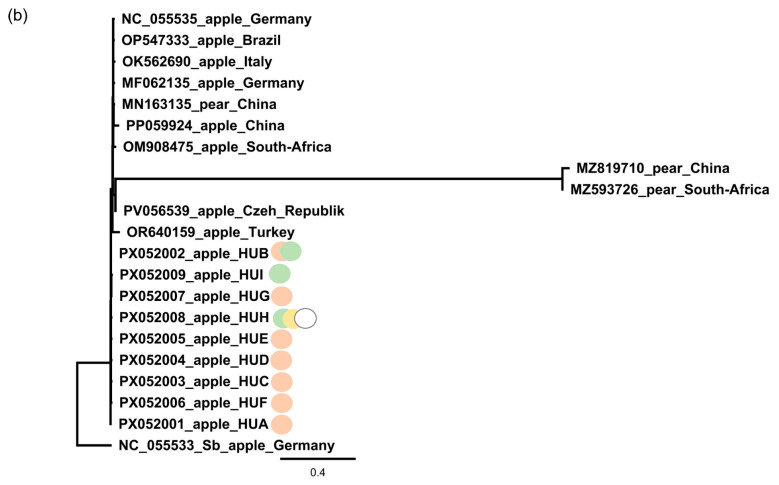
Sequence comparison of the ARWV2 partial Sa segment. (**a**) Multiple alignment and (**b**) phylogenetic analysis of the sequence of the Hungarian variants and the variants present in the NCBI GenBank (see Table S6 for their origin and details). For the multiple alignment MUSCLE algorithm, for the phylogenetic analysis, the neighbour joining method with the Tamura Nei model, with 1000 bootstrap, as a tool in Geneious Prime, was used. The reference sequence of the ARWV2 Sb (NC_055533) was used as an outgroup. The dots mark the HU variants: orange from apple, green from pear, yellow from quince, while white from the rootstock hosts.

Indels, characteristic of the pear variants, were also present in the S segment (Figure 7a), but interestingly were not present in the variants sequenced from pear in this study (Figure 7b), suggesting that their presence is not a requirement for the possibility of pear infection. The variants did not cluster according to the host species or the geographical location, indicating that they evolved after the infection in parallel.

### 3.4. The Expression of sRNAs Regulating the Lignin Pathway Has Been Observed in the ARWV2-Infected Trees

**Figure 8:**
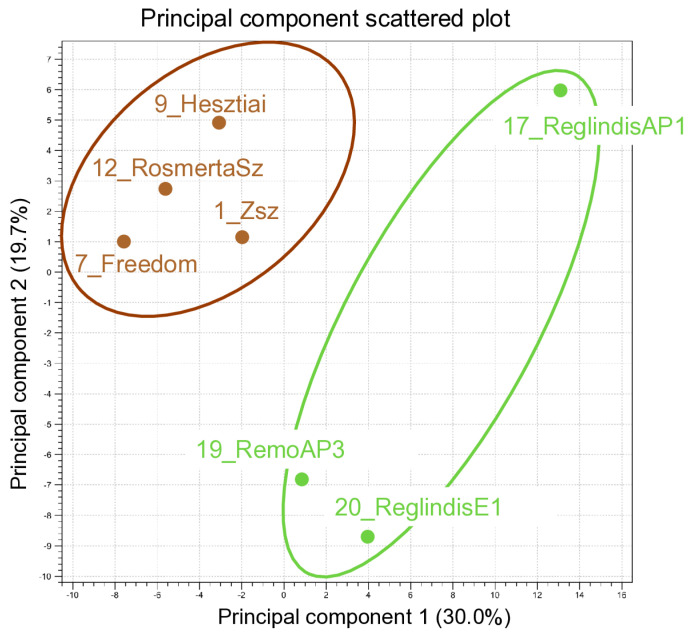
Principal component analysis of the sRNA reads sequenced in four ARWV2-infected: 1_Zsz, 7_Freedom, 9_Hesztiai, and 12_RosmertaSz (highlighted by brown colour), and in three ARWV2 non-infected: 17_ReglindisAP1, 19_RemoAP3, and 20_ReglindisE1 libraries (highlighted by green colour).

We used sRNA HTS for virus diagnostics, which enables us to investigate the changes in the host sRNA network during ARWV2 infection. For this analysis, the sRNA reads of four ARWV2-infected and three non-infected libraries were selected. sRNA HTS turned out not to be an ideal method for detecting ARWV2 infection, which is why the selection was not straightforward. The four libraries representing the ARWV2-infected trees were: 1_Zsz, 7_Freedom, 9_Hesztia, and 12-Rosmerta, because in these libraries we could detect the ARWV2 infection using RT-PCR, and the number of ARWV2-derived sRNA reads was relatively high (89–580 RPM). As the ARWV2 non-infected libraries, 17_ReglindisAP1, 19_RemoAP3, and 20_ReglindisE1 were chosen, because in these libraries the number of the ARWV2-derived sRNAs was very low (8–87 RPM) and we could not detect the virus using RT-PCR. The selected, infected trees grew at different geographical locations, under different environmental conditions, and they have been infected with several different apple-infecting viruses (Table A1), which could also affect the sRNA-ome of the host. Aware of this, first we did PCA and found that the chosen ARWV2-infected and non-infected trees clustered separately (Figure 8), suggesting the existence of important changes as an impact of the ARWV2 infection, which we aimed to detect.

Focusing only on the main differences, which could be related to the presence of ARWV2, we collected the miRNAs whose expression showed considerable changes between the two groups, were present in higher than 10 reads, and the change was characterised by a *p*-value lower than 10^−3^. As a result, we had a list of 12 *M. domestica* miRNAs, which showed up- or downregulation in the ARWV2-infected samples (Table 1).

Among them, reads of miR7123a and miR171o were present in less than 10 copies, so we did not investigate their putative role further. We found that the expression of miR398b, miR408a and b, miR397a, miR7125, and miR10996a was significantly higher, while the expression of miR482a and miR396b, miR1566t, miR10982a, and miR399k was significantly lower in the ARWV2-infected trees. The biggest changes could be observed in the increase in miR398 and miR408, and the decrease in miR399k. Altering the expression of several different transcription factors, these miRNAs regulate different biological processes, but their exact role has not been revealed in apple in detail so far. Moreover, miR408, miR397 regulate laccase, and miR7125 regulates cinnamoyl-coenzyme A reductase gene, functioning on the lignin synthetic pathway. We have not found a proper description for the targets of miR10996a, miR10982q, and miR399k, so we searched for their target by psRNA TargetFinder (Table S8b) and found that they also regulate several different transcription factors, but no correlation between the cell wall and lignin biosynthesis has been found.

**Figure 9:**
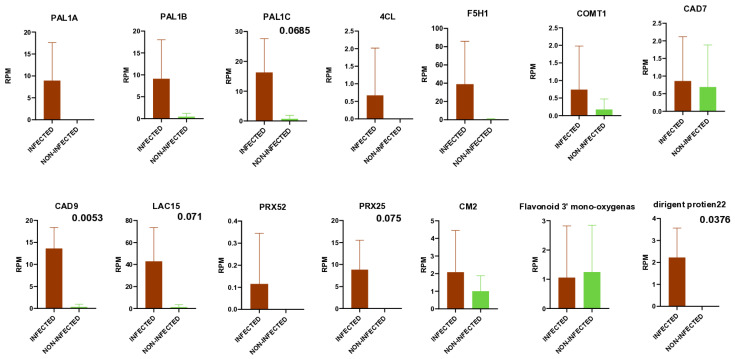
Column diagram representing RPM of the sRNAs that were mapped to the genes, encoding different enzymes on the phenylpropanoid biosynthetic pathway. Statistical analysis was performed with GraphPad Prism 10. (Free version). The quantified data were analysed using one-way ANOVA sRNAs of four ARWV2-infected and three non-infected samples were compared. PAL-phenylalanine ammonia lyase; 4CL-4-coumarate-CoA ligase; F5H1-ferulate 5-hydrolase 1; COMT1-caffeic acid 3-O-metyltransferase 1; CAD-cinnamyl alcohol dehydrogenase; LAC-laccase; PRX-peroxidase; CM-chorismate mutase. *p*-values lower than 0.1 are indicated.

Gene expression changes in symptomatic ARWV2-infected trees have been investigated by Allen and colleagues [16]. Based on RNA sequencing, they identified changes in the expression of genes functioning on the phenylpropanoid pathway. Based on the result of sRNA HTS, they have not found any ARWV2-derived sRNAs, which could be directly attributed to the inhibition of this pathway. However, upregulation of the virus-activated small interfering (vasi) RNAs mapped to the PAL gene was identified, and downregulation of the lignin biosynthetic pathway by them through an unknown mechanism has been suggested. To test the presence of this type of regulation, we mapped the sRNAs to the genes encoding key enzymes of the phenylpropanoid pathway, whose altered expression during ARWV2 infection has been identified in the above-mentioned research. Mapping ARWV2-mapped vsiRNA reads to these genes showed that there are only negligible counts of reads (max 1) targeting them (Table S9a). RPM of the not ARWV2-derived sRNAs targeting these 14 genes showed a striking difference between the ARWV2-infected and non-infected libraries (Table S9 and Figure 9).

RPM of the sRNAs targeting the phenylpropanoid pathway was always higher in the ARWV2-infected libraries, but this difference was statistically significant (*p*-value lower than 0.05) only in the case of CAD9 and dirigent protein 22. Additionally, *p*-value was 0.068, 0.07, and 0.075 in the case of PAL1C, LAC16, and PRX25, suggesting that changes in their expression can also be important during the ARWV2 infection.

**Figure 10:**
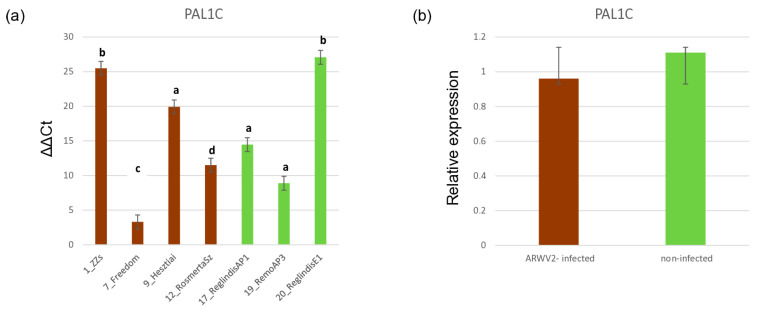
Bar graphs, representing the PAL1C gene expression of the ARWV2-infected and non-infected trees. (**a**) ΔΔCt values using qRT-PCR, detecting *M. domestica* expression using actin and ubiquitin as controls, was used to calculate (**b**) relative expression of PAL1C in the ARWV2-infected and non-infected trees. For individual trees, three technical replicates were used. Data are presented as the mean ± SD (*n* = 3). Statistical analysis was performed via one-way ANOVA. Post hoc analysis was performed using Tukey’s test and is marked using a compact letter display (CLD). The samples labelled with the same letter were not significantly different (significance level 0.05) from each other.

sRNA-targeted downregulation of the PAL has been detected previously, when Allen and colleagues used RNAseq to quantify the PAL level [16]. We have found three PAL1 in the *M. domestica* genome, and found significantly more sRNAs targeting PAL1C in the infected libraries. To see how this upregulation affects the PAL1C expression in the ARWV2-infected trees, the expression level of PAL1C was tested in the samples that we used for the sRNA expression analysis (Figure 10).

Although as on average, the PAL1C expression in the ARWV2-infected trees decreased, we found lower PAL1C expression only in the 7_Freedom tree, while in the other ones, the PAL1C level did not correlate with the absence or presence of the virus.

## 4. Discussion

In our previous study, we looked for the presence of viruses in apple trees growing at germplasm collections and production orchards using sRNA HTS [19]. As a result, the frequent presence of ALV-1 and CCGaV has been revealed and was validated using RT-PCR. Comparing the results of the detailed bioinformatic analysis and its validation suggested that while RNA-seq can reliably detect the presence of these viruses, sRNA HTS diagnostics for viruses infecting apple could miss their detection. This conclusion has been drawn from a collective proficiency test study conducted previously [24], and this is what we experienced in this study.

Recently, the presence of ARWV1 and ARWV2 has been described in the Czech Republic, where the initial analysis failed to detect the presence of these viruses [14]. This is why we started to search for their presence in our original sRNA datasets and found an indication of the presence of ARWV2 in some of them.

In agreement with previous studies [25], we found a contradiction between the sRNA HTS and its RT-PCR validation in several cases. sRNA HTS using a stringent threshold detected ARWV2 presence in five libraries, while when using a permissive threshold, its presence was suggested in nine additional libraries. RT-PCR using diagnostic primers targeting Sa segments failed to detect the virus in eight out of the positive samples. The RT-PCR was ARVW2-positive in seven samples, including 9_Hesztiai and 21_RemoE2, in which the sRNA HTS did not detect the virus. ARWV2 is a very variable virus; its sequence contains long stretches of repetitive elements, why optimal primer design a challenge for this virus. Because of sequence differences within the primer annealing part of the genome, variants can escape from the RT-PCR diagnostics, explaining why the currently available diagnostic test frequently gives inconsistent results [25]. The possibility of reassortment of the viral segments and recombination could further increase the presence of variants and make their diagnostics more difficult. We think that ARWV2 was present and could have been detected in most of our samples if further optimisation of the primers had been carried out, which we plan to do in the future.

RT-PCR validation of the L and Ma segments was only successful in the case of the 7_Freedom library. Because of only partial coverage of the viral genome by sRNAs, the efficiency of the primer annealing could be tested only in this library, as sRNA reads in the other ones did not map to this part of the viral segments. Although there was a meaningful coverage of these segments and RPM of these segments-derived sRNA reads in all but three libraries, we could not amplify them. The reason for this could be that i/the primers used in the PCR reaction failed to anneal to the template, and/or ii/ the titre of the L and Ma segments in these samples was below the detection limit.

ARWV2 level in the infected tree can vary and can drop below the detection limit, which further increases the unreliability of the RT-PCR-based diagnostic tests. Presence of ARWV in the propagation material is usually tested by a three-year biotest using Lord Lambourne indicator. The ten cultivars, which were diagnosed as a pool in the 16_apple library, were in parallel tested by this method and found to be ARWD-free, suggesting that if the virus is present at a low level in the branch of the mother tree that is used for the bud grafting, the virus can remain undetected.

In some cases, we sampled the same tree more than once. Trees included in the 16_apple library were tested both in 2017 and in 2019. While we found Ozak Gold ARWV2-infected in both years, in 2017, the Regal Prince, while in 2019, the Jonica cultivar was found to be ARWV2-infected. Reglindis and Remo cultivars growing in Olcsvaapáti were also tested twice in 2017 and in 2020. While at first, Reglindis AP2 and Remo E2 were ARWV2 infected, in 2020, in addition to Remo E2, we detected the virus in Reglindis E1 and in an additional Reglindis tree, but not in Reglindis AP2. This finding suggests that the variant can be detected, and the reason for the negative test is more likely the low titre of the virus, which can vary over the years. Global warming and climate change can also affect the condition of the tree and the ARWV2 titre.

We used sRNA HTS for virus diagnostics in woody plants for a long time and found a contradiction between sRNA HTS and RT-PCR diagnostics in several cases. Long-lasting viral infection in woody plants could result in the decreased amplitude of the sRNA RNAi response, as we experienced this in the case of grapevine rupestris stem pitting-associated virus and grapevine virus T infection in grapevine [26,27], and we assume that it could be the case in the ARWV2-infected apple trees, which are several decades old.

Sequence comparison and multiple alignment of the ARWV2 HU variants showed that it shares more than 96% identity with variants originating from different parts of the world (Table S7). ARWV2 variants from pear host cluster separately in the case of the L and M segment (Figure 5 and Figure 6), but in the case of S segments variants originating from pear hosts clustered together with the variants originating from the apple host (Figure 7b), suggesting that the clustering, according to the host, is not the cause of the different clustering.

The origin of the ARWV2 infection in Hungary is unknown. Some of the ARWV2 positive trees: Nyari fontos, Berecki, Vilmos, and Piros Vilmos are Hungarian cultivars which could only be infected at the stock collections, by unknown vectors, or by the use of ARWV2-infected rootstock. As we have found ARWV2 infection in the rootstock itself, this possibility seems valid.

Investigating the capability of the sRNAs, showing altered expression during ARWV2 infection to target the genes of the phenylpropanoid pathway, we found that only the ARWV2 unmapped reads showed alteration. Their expression increased, which could, in theory, alter the gene expression pattern of the genes and consequently the activity of the enzymes of this pathway. To test how the increased number of mapped sRNAs to a target gene affects its expression, the relative expression of one of the PAL genes (PAL1C) was measured using qRT-PCR, but no statistically relevant decrease in its level in the ARWV2-infected trees has been found. Allen and colleagues found a nice correlation with the lignin in the xylem and gene-expression pattern of the enzymes on the phenylpropanoid pathway, in the infected trees, but they investigated very young (1-year-old), symptomatic trees [16]. In our case, we tested and surveyed 30–40-year-old, asymptomatic trees, which could be the reason why we did not obtain statistically significant differences in the PAL expression between the ARWV2-infected and non-infected trees. However, we have found an increase in the number of the sRNAs that target not only PAL1, but also other key enzymes of the pathway, suggesting that this slight alteration and inhibition of the pathway could happen in parallel, which could have an additional effect on the lignin content of the plant. To answer the question of how the altered miRNA profile in the ARWV2-infected trees can alter the lignin content of old trees, systematic studies comparing lignin content measurements and miRNA profiling of possible only ARWV2-infected and virus-free apple trees can be carried out in the future. The miRNA-ome of the apple has been investigated intensely for a long time. While the first report used Northern blotting and in situ hybridisation for the detection of the miRNAs [28], the emergence of sRNA HTS allowed a complete and comprehensive description of the apple miRNAs [29]. This initial report has been supplemented by new miRNA members, when the miRNA profile of apple trees under different environmental and stress conditions has been investigated [30,31,32,33,34]; however, we could not find any reports in which changes in the miRNA pattern of the apple host to virus infection have been studied. The reason for this could be that it is very difficult to find suitable biological replicates of the same cultivar infected with a single virus. This has not been true even in our case; we did not have several trees of the same cultivar, infected with a single virus, growing in the same environmental conditions. The apple trees whose sRNA-ome was investigated were different cultivars, infected by different sets of viruses, and grown under different environmental conditions. From them, we chose four ARWV2-infected and three non-infected trees, which PCA analyses suggested that they are suitable representatives of the ARWV2 infection. When we compared their miRNA profile, we focused only on the miRNA changes that showed the most dramatic change with a *p*-value suggesting reliable differences between the two groups. Based on these analyses, we found dramatic changes in the expression of some of the miRNAs. The most upregulating miRNAs were members of the miR398, miR408, and miR397 families. mir398 is a regulator of plant development and stress response, and was found to be upregulated in the ARWV2-infected trees 175 times. This miRNA has been known to regulate Cu metabolism through controlling CDS1 and CDS2 [35]. Two members of the miR408 family, mdm-miR408a and mdm-miR408b, showed a 106- and 79-fold increase in the ARWV2-infected plants, respectively. miR408 controls the expression of laccase, and through its regulation, it controls antioxidant content and lignin biosynthetic processes of the cells [36]. Both miR398 and miR408 are induced during virus infection when the miRNA pattern of Papaya meleira virus-infected Papaya was described [37]. Additionally, the expression of miR397 and miR7125 has been increased by 33 and 6-fold in the ARWV2-infected plants. miR397 also targets genes in the laccase family [38]. Its level has been investigated in Botryosphaeria-infected apples and found to be directly correlated with the lignin content of the cell wall and pathogen sensitivity of the host [39]. During pathogen infection, the increased miR397 inhibited the MhLAC7 level, resulting in a decrease in the lignin content of the cell wall. Interestingly enough, investigating the expression of miR7125, targeting the cinnamoyl-coenzyme A reductase gene (CCR) in apple, revealed that its increased expression led to reduced lignin content [40]. In contrast to our work, where we found the induced level of miR7125 during ARWV2 infection, during infection with other Colletotrichum, the accumulation of the SA level promotes a decrease in the miR7125 level, which results in increased lignin content and pathogen resistance of the host [41].

In the ARWV2 infected trees, the most down-regulated miRNAs were members of the miR399, miR482, miR396, miR156, and miR10982 families. None of these miRNAs have been found to be involved in the lignin biosynthetic pathways, but they have been found to regulate the pathogen response of the host. miR482 plays a role in the pathogen recognition of the host as it regulates the expression of NBS-LRR genes [41]. Together with miR156, regulating WRKY transcription factors, its expression is found to be induced in response to *Alternaria alternata* f. sp. mali infection in apple [41,42]. In the ARWV2-infected plants, we found that both the level of miR482 and miR156 has been decreased, suggesting that virus infection alters their level differently. miR396 is a highly expressed miRNA in apples [34], targeting not only growth response factors which regulate and coordinate cell division in the meristem, but in plants also five IAA-amino acid hydrolase genes, three replicate factor C subunit 1 genes, and one TIR-NB-LRR resistance gene [29]. miR399 is a long-distance signal, regulating inorganic phosphate transmembrane transporters in apples [33]. To investigate the possible role of the above miRNAs and the alteration of their potential target gene expression in the flexible-wood phenotype, CRISPR knockout of the predictive targets and lignin-content measurement of the genome-edited trees could be carried out in the future.

## 5. Conclusions

In our work, in correlation with the reports from different geographical locations, we found ARWV2 infection in asymptomatic trees or in symptomatic trees with multiple infections, making it impossible to correlate the symptoms to the disease. The presence of the virus could induce sRNA-regulated alteration in the expression of the enzymes of the phenylpropanoid pathway, the key regulators of lignin biosynthesis; however, this effect varies with the sensitivity of the cultivar, the health of the tree, and also according to the environmental conditions. The symptoms and the virus titre of the trees could change, which could affect these uncertainties, but could be answered as a result of the recently launched long-term symptom investigation of selected, ARWV2-infected trees in Germany [25]. As the virus is widespread and the fruits of ARWV2-infected trees have been consumed for a long time, the possibility of using ARWV2-infected trees, with decreased lignin content, as an environmentally friendly biofuel production alternative could arise. Our work shows that although this strategy could work in young grafts, its outcome in the older trees is uncertain, and why this strategy as a solution for easy digestible cellulose access can be planned after detailed studies involving lignin content measurements of the ARWV2-infected trees. However, systematic characterisation of the lignin biosynthetic pathway in ARWV2-infected trees, including pear and quince, could deliver important conclusions, which could be used in the future for this purpose and would be important to plan.

## Figures and Tables

**Table 1:** List of the miRNAs whose expression showed the biggest change during the ARWV2 infection, highlighting the name and function of their potential targets. See Table S8 for details.

Name	Max RPM	Fold Change	*p*-Value	Potential Target	Function of the Target
mdm-miR398b	367,679	175.10	3.07557 × 10^−7^	CDS1 and CDS2	Cu metabolism, role in proteosomal degradation
mdm-miR408a	9479	106.66	8.16911 × 10^−6^	peptide chain release factor, laccase	regulation of the antioxidant content of the cell
mdm-miR408b	12,200	79.27	8.07007 × 10^−6^	peptide chain release factor, laccase	
mdm-miR397a	2067	33.73	7.42754 × 10^−5^	target the laccase (*LAC*) genes	lignin synthesis
mdm-miR7125	400,675	6.33	0.004234914	cinnamoyl-coenzyme A reductase gene (CCR)	biosynthesis of lignin and anthocyanins in response to light and salicylic acid (SA) signals
mdm-miR10996a	59,825	6.25	0.002462787	transcription factor bHLH94-like	
mdm-miR482d	148	−4.76	0.0023785	NBS-LRR genes	pathogen recognition
mdm-miR396b	12,176	−5.09	0.002962458	GRF growth responsive factor	coordination of cell division
mdm-miR156t	260	−7.25	0.005670341	SQUAMOSA-PROMOTER BINDING PROTEIN-LIKE (SPL)	transition from vegetative to reproductive growth
mdm-miR7123a	8	−10.04	0.005860199		
mdm-miR10982a	17	−12.01	0.000976011	homeobox-leucine zipper protein HDG11-like	
mdm-miR482a-5p	8586	−27.94	2.65576 × 10^−6^	NBS-LRR genes	pathogen recognition
mdm-miR171o	2	−41.17	5.52816 × 10^−5^		
mdm-miR399k	77	−291.31	0	ubiquitin-conjugating enzyme and inorganic phosphate transporter 8-like	long-distance signal for regulators

## Data Availability

The original contributions presented in this study are included in the article/Supplementary Materials. Further inquiries can be directed to the corresponding author. HTS datasets have been archived in the NCBI GEO: GSE205183, GSE306190. Sanger sequences of the ARWV2 variants can be accessed at the NCBI GeneBank: PX052001-PX052011.

## Supplementary Materials

The following supporting information can be downloaded at: https://www.mdpi.com/article/10.3390/v17101394/s1, Figure S1. Location of the sampling on the map of Hungary; Figure S2. Size distribution of the ARWV2-mapped small RNAs in the sequenced libraries; Table S1. Information on the samples, which were tested for the presence of ARWV2 in the survey. Table S2. Initial statistics of the sequenced small RNA libraries; Table S3. Detailed results of the bioinformatics analysis of small RNA HTS of different libraries for ARWV2; Table S4. Sequences of the PCR primers used for (a) virus detection, or for (b) functional analysis of the endogenous genes with their appropriate references; Table S5. List of ARWV2-derived sequences which were deposited into the NCBI GenBank; Table S6. Virus records of ARWV2 in the NCBI, highlighting the sequences which were used in the Phylogenetic analyses; Table S7. Percent identity matrix of the ARWV2 partial A/L, B/M, and C/S segment sequences used in the phylogenetic analysis; Table S8. List of the miRNAs whose expression showed the biggest change during the ARWV2 infection; Table S9. Comparison of the expression of sRNAs of the ARWV2-infected and non-infected libraries, targeting the genes on the phenylpropanoid pathway.

## Appendix A

**Table A1:** Detailed information about the apple samples that were investigated using small RNA HTS.

Small RNA Library Name	Place and Type of Sampling			Cultivar/ Variety	Year of Sampling	Viruses Identified by RT-PCR
1_Zsz	**Production gardens**	**orchard**	Zalaszanto	Fuji	2015	ACLSV, ASPV, ASGV, APMV, **ARWV2**
2_SZH			Tamasi	non identified		ASGV
3_SS			Soroksar	non identified		ASGV
4_idared			Vamosmikola	Idared		AHVd
5_golden			Vamosmikola	Golden D		ASGV, CCGaV
6_JG			Vamosmikola	Jonagold		ACLSV, ASPV, ASGV, APMV, CCGaV, AHVd
7_Freedom			Ujfeherto	Freedom		CCGaV, AHVd, **ARWV2**
8_RosmertaI	**Germplasm**	**mother trees in an isolator house own roots**	**Ujfeherto**	Rosmerta	2015	AHVd
9_HesztiaI				Hesztia		ACLSV, ASPV, ASGV, APMV, AHVd, ALV-1, **ARWV2**
10_ArtemiszI				Artemisz		ACLSV, ASPV, ASGV, APMV
11_CordeliaI				Cordelia		ACLSV, ASPV, AHVd
12_RosmertaSz		**mother trees in an open field**	**Germplasm collection at Soroksar**	Rosmerta		ACLSV, ASPV, ASGV, APMV, **ARWV2**
13_HesztiaSz				Hesztia		ACLSV, ASPV, ASGV, APMV, AHVd
14_ArtemiszSz				Artemisz		ASPV, ASGV, APMV, ALV-1, CCGaV
15_CordeliaSz				Cordelia		ACLSV, ASPV, APMV, AHVd, ALV-1
16_apple	**Germplasm**	**old certified stock, M4 or M26 rootstock**	**Erd/Elvira**	Ozark gold	2017	AHVd, ALV-1, **ARWV2**
				Jonagold		AHVd, ALV-1
				Florina		CCGaV
				Jim Brian		ACLCV, ASPV, AHVd, ALV-1
				Jeasymac		AHVd, ALV-1
				Jonica		ACLSV, ASPV, ASGV
				Red Elstar		AHVd, ALV-1
				Regal prince		AHVd, ALV-1, **ARWV2**
				Redwinter		ACLCV, ASPV, AHVd, ALV-1
				Top spur		ALV-1
17_ReglindisAP1	**Production gardens**	**open field plantation**	**Olcsvaapáti**	Reglindis AP1	2017	ASGV, AHVd, ALV1, CCGaV
18_ReglindisAP2				Reglindis AP2		ASGV, AHVd, CCGaV, **ARWV2**
19_RemoAP3				Remo AP3		AHVd
20_ReglindisE1				Reglindis E1		ASGV, AHVd, CCGaV
21_RemoE2				Remo E2		AHVd, **ARWV2**
22_RemoE3				Remo E3		ALV-1
