# Supplementary material for: Investigating Apple Rubbery Wood Virus 2: HTS-Based Detection in Hungary and Involvement of sRNA-Based Regulation Processes During Its Infection
Source: Viruses. 2025 Oct 20;17(10):1394. doi: 10.3390/v17101394 (PMC12568118; doi:10.3390/v17101394)
Supplement: Supplementary file 1 [file viruses-17-01394-s001.zip › viruses-3871230-supplementary-figures.pdf]

viruses

MDPI

Article

Investigating Apple Rubbery Wood Virus 2: HTS-Based Detection in Hungary and Involvement of sRNA-Based Regulation Processes During Its Infection

Almash Jahan and Éva Várallyay *

Genomics Research Group, Department of Plant Pathology, Institute of Plant Protection, Hungarian University of Agriculture and Life Sciences, Szent-Györgyi Albert Street 4, 2100 Gödöllő, Hungary; almashjahan010@gmail.com
* Correspondence: varallyay.eva@uni-mate.hu

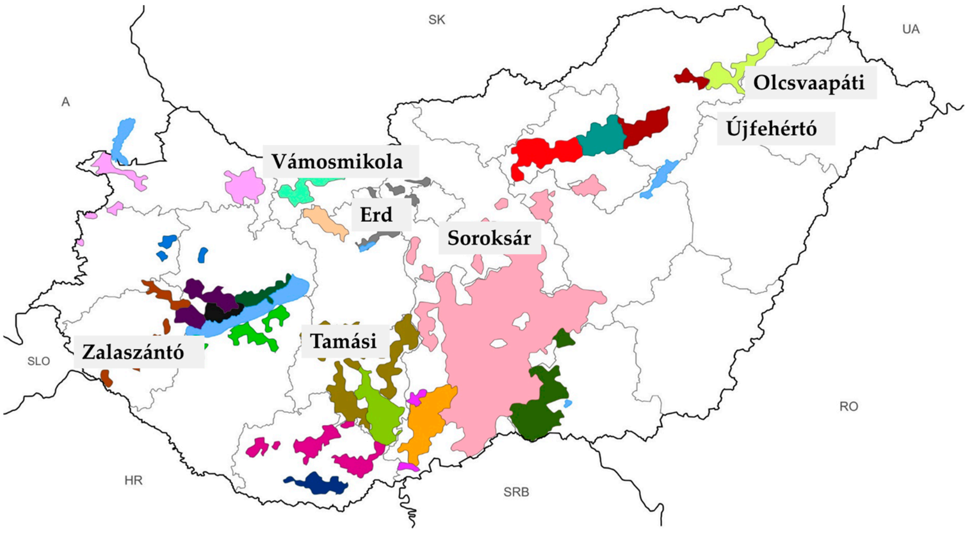

Figure S1. Location of the sampling on the map of Hungary.

 https://doi.org/10.3390/v17101394

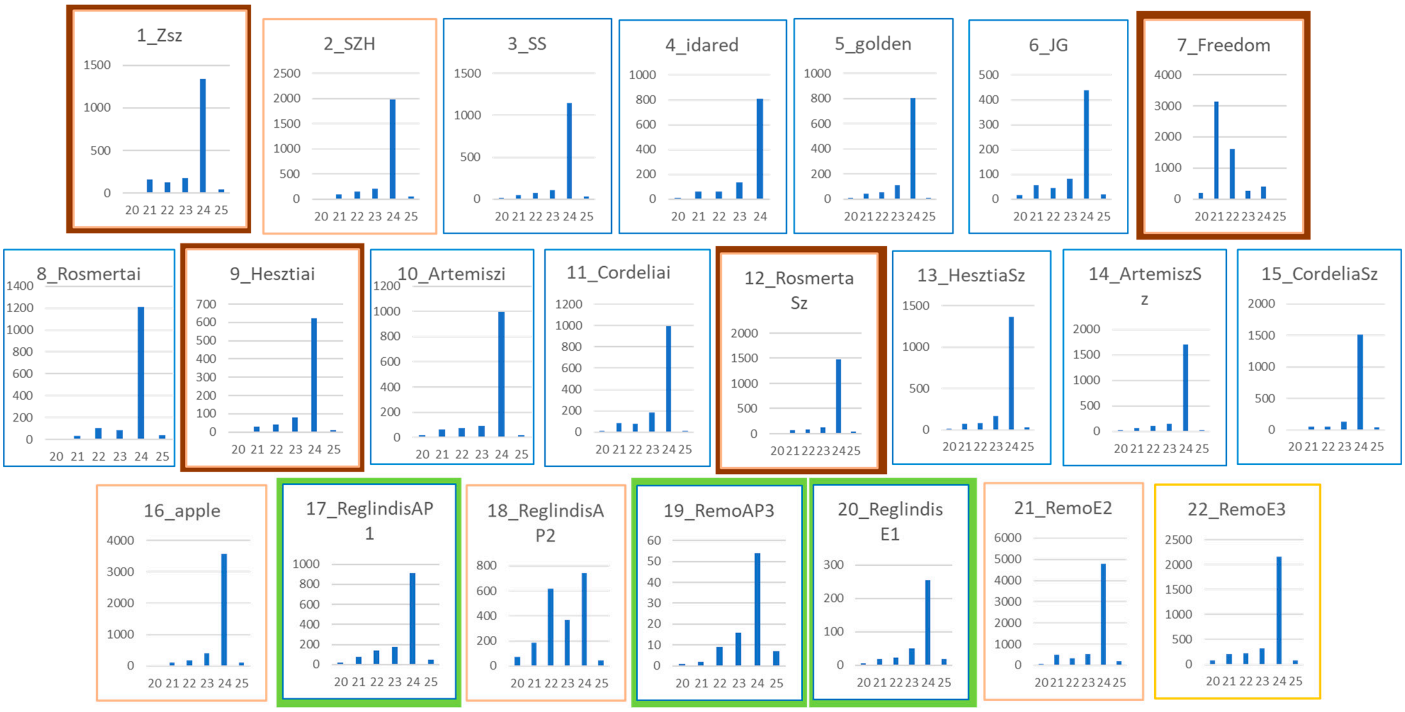

Figure S2. Size distribution of the ARWV2-mapped small RNAs in the sequenced libraries. Orange colour marks, the samples in which ARWV2 could be detected by RT-PCR. Brown and green boxes indicate the lARWV2-infected and non-infected libraries, respectively, which were used for the small RNA expression analysis.
